# Amount and intensity of daily living activities in Charcot–Marie–Tooth 1A patients

**DOI:** 10.1002/brb3.187

**Published:** 2013-10-30

**Authors:** Federica Menotti, Luca Laudani, Antonello Damiani, Andrea Macaluso

**Affiliations:** 1 Department of Human Movement, Social and Health Sciences, University of Rome Foro ItalicoRome, Italy; 2 Sezione Laziale, Unione Italiana Lotta alla Distrofia Muscolare (UILDM)Rome, Italy

**Keywords:** Energy expenditure, hereditary neuromuscular disorder, neuropathies, rehabilitation, speed of walking

## Abstract

**Background:**

Charcot–Marie–Tooth 1A (CMT1A) patients show a reduction of spontaneous activities of daily living measured by means of questionnaires or pedometers, which are quite inaccurate compared to recent measurement techniques.

**Aim:**

The study aimed at quantifying daily living activities in CMT1A patients by means of inertial sensors, which give information not only on the amount but also on the intensity of these activities.

**Materials and methods:**

Time and count (amount), and velocity and power (intensity) of 24 h daily living activities were measured in eight patients (20–48 years; Barthel >90; Tinetti >20) and eight healthy individuals, matched for age and gender, by means of a wearable inertial sensor device.

**Results:**

There were no differences between patients and controls in the 24-h distance covered and count of steps. However, count of step climbing and sit to stand were lower in patients than in controls (139.93 ± 141.66 vs. 341.06 ± 164.07 *n* and 58.23 ± 7.82 vs. 65.81 ± 4.75 *n*, respectively; *P *<* *0.05) as well as mean daily step-climbing and walking velocities (1.07 ± 0.17 vs. 1.21 ± 0.10 m/sec and 1.16 ± 0.31 vs. 1.87 ± 0.50 m/sec, respectively; *P *<* *0.05). In CMT1A patients there was a positive correlation between strength of the knee extensor muscles and both count of steps climbed (*R *=* *0.80) and sit to stand (*R *=* *0.79).

**Discussion and conclusion:**

The reduced ability of CMT1A patients to carry out activities at high intensity, which was correlated with strength, suggests that strength training might be a rehabilitation tool for improving the 1 ability to carry out these activities.

## Introduction

Charcot–Marie–Tooth 1A disease (CMT1A), also referred to as hereditary motor and sensory neuropathy (HMSN1A), is a genetic and progressive neuropathy affecting the neuromuscular system (Casasnovas et al. 2008; Banchs et al. 2009). Patients with CMT1A are affected by segmental demyelination of peripheral nerves, reduction in the nerve conduction velocity, and consequent axonal degeneration that impair functions of the distal part of legs and arms (Krajewski et al. 2000; Hattori et al. 2003).

Previous studies reported CMT1A patients with several functional limitations: muscle weakness or atrophy in both upper and lower limbs (Lindeman et al. 1999; Menotti et al. 2012), high level of experienced fatigue and impaired recovery from fatigue after exhausting motor tasks (Schillings et al. 2007; Zwarts et al. 2008; Menotti et al. 2012), modification of walking patterns (Don et al. 2007), high energy cost of walking (Menotti et al. 2011, 2013), and low aerobic capacity and cardiovascular fitness (Wright et al. 1996; Fowler 2002; Kilmer 2002; El Mhandi et al. 2008).

Most of these functional limitations are the result of the progression of the neuropathy itself, but can also be exacerbated by a sedentary lifestyle. However, to the best of the authors' knowledge, there are no quantitative data on the amount of daily living activities in CMT1A patients. In a few studies carried out on mixed groups of patients with various neuromuscular disorders, including CMT1A patients, it has been reported as a reduction in spontaneous activities of daily living measured by means of questionnaires (Aitkens et al. 2005) or pedometers (Kilmer et al. 2005), which are quite inaccurate compared to recent measurement techniques based on inertial sensors. Inertial sensors have been demonstrated to be valid and reliable methods to assess not only the amount of daily living activities (number of steps, total distance, walking time) but also the intensity at which these activities are carried out (speed and power of walking, running, jumping, and step climbing) (Benedetti et al. 2009; Kwon et al. 2010). Moreover, the relationship between both the amount and intensity of activities of daily living and neuromuscular function in CMT1A patients is currently unknown.

Therefore, the aim of this study was to compare the amount and intensity of activities of daily living, measured by means of inertial sensors, between patients with CMT1A and healthy individuals. A second aim of the study was to look at the association between both amount and intensity of activities of daily living and muscle strength, which is one of the major determinants of functional limitations. It was hypothesized that CMT1A patients carried out a lower amount and intensity of activities of daily living with respect to healthy individuals and that, in CMT1A patients, patterns of activities of daily living correlated with muscle strength.

## Material and Methods

### Participants

Eight patients with CMT1A (three male and five female individuals; mean age 35.9 ± 9.9 years, age range 20–48 years; mean body mass 67.6 ± 10.6 kg) and eight healthy adults (three male and five female individuals; mean age 35.1 ± 11.2 years, age range 21–50 years; mean body mass 67.6 ± 10.1 kg) participated in the study. Volunteers with CMT1A were recruited from the UILDM Rehabilitation Centre in Rome. The inclusion criteria were as follows: (1) diagnosis of CMT1A by genetic test; (2) Barthel index >80 (Jacelon 1986) and Tinetti score >20 (Tinetti 1986); (3) age between 20 and 50 years; and (4) no clinical signs of heart or pulmonary disease. A consultant neurologist attributed to all patients the CMT neuropathy score (Shy et al. 2005). Muscle strength of upper and lower limbs was assessed according to the Medical Research Council (MRC) scale (Medical Research Council 1976). Selected patients had a mean Barthel Index of 96.3 ± 3.8 (mean ± SD); a Tinetti score of 22.3/28 ± 2.6 (mean ± SD); a CMT neuropathy score of 13.25 ± 3.41 (mean ± SD); a CMT neuropathy score range 8–16; a mean MRC score of upper limbs of 61.75/70 ± 5.90 (mean ± SD) assessed on deltoid, biceps brachii, triceps brachii, extensor digitorum, interosseus, lumbricalis, and abductor pollicis muscles; and a mean MRC score of lower limbs of 76/90 ± 9.37 (mean ± SD) assessed on gluteus maximum, gluteus medium, quadriceps, hamstrings, ileopsoas, triceps surae, tibialis anterior, peroneus, and extensor hallucis longus muscles. All patients were able to walk without walking aids or ankle-foot orthoses (AFOs) and were functionally independent and fully integrated in activities of daily living.

The individuals of the control group were selected from the employers of the rehabilitation center and were matched to the CMT patients for age, gender, and body mass. With Ethics approval, the study was carried out in accordance with the Declaration of Helsinki and informed consent was obtained from all participants.

### Instrumentation and measurements

Daily physical activities were measured by means of an inertial sensor system (IDEEA, Intelligent Device for Energy Expenditure and Activity; MinisunLLC, Fresno, CA), which is a portable device, 75 × 55 × 15 mm in size, worn at the waist. Five miniaturized sensors (16 × 14 × 6 mm) were taped to the body as follows: one on the front of the chest, one on the front of each thigh, and one on each sole. In addition, three electrodes for electrocardiography (ECG) were placed on the volunteers' chest. The five sensors provided continuous signals of angle, relative position and acceleration, whereas ECG electrodes provided hearth rate signals. All signals were sent through a thin wire to a microprocessor in the device that saved the information on a flash memory card and sampled at 32 Hz.

Participants wore the IDEEA device in two recording sessions of 24 h each, with an interval of 1 week between sessions. During the recording sessions volunteers were asked to carry out activities of their usual daily life. Data from the device were downloaded into a peripheral computer (Sony Vaio VGN-S5XP/B; Sony Europe, Surrey, U.K.) for further analysis.

Measurements of muscle strength were carried out in the laboratory on a separate day. Participants were asked to seat on a custom-made chair and stabilized by a waist belt. The chair was upright positioned and both hip and knee angles were at 90°. The frontal side of the ankle was in contact with a support linked to a fixed force transducer. The position allowed participants to exert isometric contractions of the knee extensors muscles in the sagittal plane. Torque of both elbow flexor and knee extensor muscles of the dominant limb was measured by a force transducer (Kistler 9203; Winterthur, Switzerland). The force signal was amplified (x1K) (Kistler Charge Amplifier Type 5011), displayed on a oscilloscope in front of the participants (Tectronix TDS 220; Beaverton, WA), and then stored on a PC laptop (Sony Vaio VGN-S5XP/B; Sony Europe), with a sampling frequency of 2048 Hz, using a PCMCIA card (A/D NI DAQ 6024E; National Instruments, Austin, TX).

To record maximal isometric voluntary contraction (MVC) participants were able to follow their performance on the oscilloscope and were verbally encouraged to achieve a maximum and to maintain it for at least 2–3 sec before relaxing. Three attempts were performed, separated by 5 min, and the greatest of the three attempts was chosen as MVC (Macaluso and De Vito 2003).

### Data analysis

Data recorded by the inertial sensor system were analyzed off-line with algorithms and software to interpret the type of posture changes and body motion such as the onset, duration, and frequency of these activities (MiniSun GaitView 2 2.2). Amount of daily living activities was expressed in terms of daily energy expenditure (kcal), daily walking distance (m), time (min), and count (*n*) of each activity (sitting, reclining, lying, walking, running, jumping, and step climbing). The time spent resting (min) was obtained by summing the time of sitting, reclining, and lying. Time and count of sit to stand were calculated as the sum of transitions between sitting (or reclining) and standing. Time and count of stand to sit were obtained as the sum of transitions between standing and sitting (or reclining). Time and count of transition were obtained as the sum of sit to stand and stand to sit. Intensity of daily living activities was expressed as speed (m/sec) and power (W) of walking, running, jumping, and step climbing. All data are presented as the average of the two 24-h sessions.

Mechanical data were analyzed off-line using LabVIEW 8.0 Software (National Instruments). Torque was calculated as the product of the force recorded by the transducer and the distance between the axis of rotation of the joint and the point where force was applied. MVC torque was chosen as the mean value of a 1-sec window around peak torque.

### Statistics

All data were normally distributed in terms of skewness and kurtosis (all values <2). Statistical comparisons of each parameter (energy expenditure, walking distance, resting time, walking time, running time, jumping time, step-climbing time, sit-to-stand time, stand-to-sit time, transitions time, walking count, running count, jumping count, step-climbing count, sit-to-stand count, stand-to-sit count, transitions count, walking speed, running speed, jumping speed, step-climbing speed, walking power, running power, jumping power, and step-climbing power), between groups (patients and individuals of the control group) were carried out using a two-sample Student's *t*-test. Pearson correlation coefficient was calculated to look at the association between each parameter from IDEEA and muscle strength. Statistical significance levels were set at *P* < 0.05. Unless otherwise specified, data were presented as mean ± standard error of the mean.

## Results

### Amount of physical activity

There were no significant differences in the mean energy expenditure of the 24-h sessions between CMT1A patients (2437.59 ± 353.69 kcal) and healthy controls (2361.32 ± 515.86 kcal). Similarly, walking distance did not differ between groups (4573 ± 2949 m for CMT1A patients and 4759 ± 1259 m for healthy controls).

Time and count of daily activities in both CMT1A patients and healthy controls are reported in Table 1. There were no significant differences between patients and individuals of the control group in either time or count of resting, walking, running, and jumping.

**Table 1:** Time, count, speed, and power of resting, walking, running, and jumping (mean ± SD) in patients and control group

	Time, min (mean ± SD)		Count, *n* (mean ± SD)		Speed, m/sec (mean ± SD)		Power, W (mean ± SD)	
	Patients	Controls	Patients	Controls	Patients	Controls	Patients	Controls
Resting	984.52 ± 107.09	1023.60 ± 20.92	–	–	–	–	–	–
Walking	71.08 ± 43.66	66.91 ± 15.17	6254.07 ± 3845.85	6825.25 ± 1825.47	55.99 ± 7.50*	65.70 ± 4.45	48.76 ± 6.98	51.45 ± 10.33
Running	0.65 ± 1.48	1.54 ± 3.01	11.93 ± 24.55	130.43 ± 190.43	2.19 ± 0.07	2.46 ± 0.53	102.17 ± 22.97	129.70 ± 54.43
Jumping	0.029 ± 0.05	0.065 ± 0.09	3.29 ± 7.02	13.14 ± 15.31	1.56 ± 0.20	1.75 ± 0.15	122.57 ± 23.13	136.34 ± 38.80

* Significantly different from control group (*P* < 0.05).

**Figure 1:**
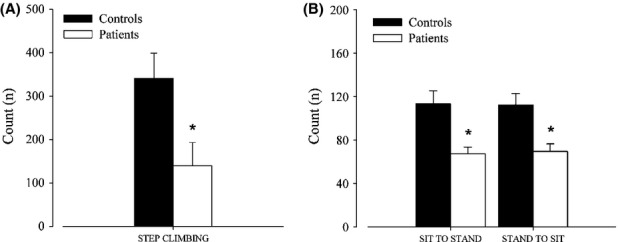
(A) Step-climbing count (mean ± SE) and (B) sit-to-stand and stand-to-sit count (mean ± SE) in patients and healthy individuals of the control group. *Significantly different from control group (*P* < 0.05).

Time of step climbing did not differ between CMT1A patients (2.42 ± 2.60 min) and individuals of the control group (2.97 ± 1.25 min), whereas count of step climbing was significantly lower in CMT1A patients with respect to controls as showed in Figure 1A. Similarly, count of sit to stand and stand to sit was significantly lower in CMT1A patients with respect to controls, as showed in Figure 1B, and time of both activities was significantly lower in CMT1A patients than controls (sit to stand: 1.12 ± 0.28 min in CMT1A patients and 1.89 ± 0.56 min in controls; stand to sit: 1.14 ± 0.31 min in CMT1A patients and 1.87 ± 0.50 min in controls, *P* < 0.05).

### Intensity of physical activity

The statistical analysis showed that mean speed of walking was significantly lower in CMT1A patients with respect to individuals of the control group, whereas power was not statistically different between the two groups as reported in Table 1.

**Figure 2:**
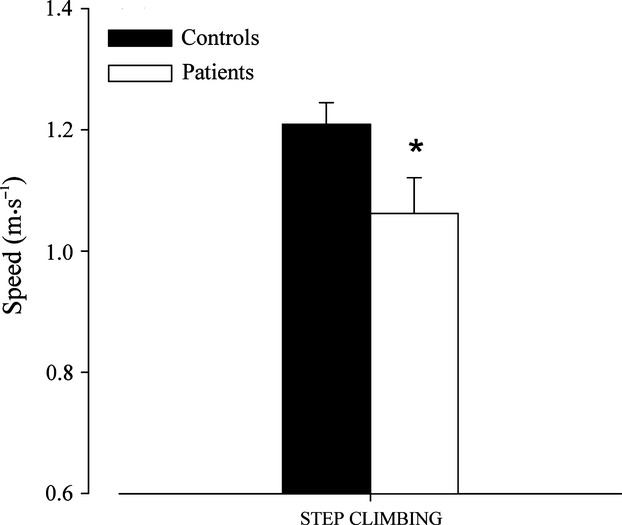
Step-climbing speed (mean ± SE) in patients and healthy individuals of the control group. *Significantly different from control group (*P* < 0.05).

In addition, step-climbing speed was statistically lower in CMT1A patients with respect to individuals of the control group (Fig. 2), whereas power was not statistically different between the two groups (112.79 ± 12.6 W for CMT1A patients and 127.76 ± 22.99 W for controls).

Speed and power of CMT1A patients and healthy controls in running and jumping are reported in Table 1. There were no significant differences between patients and individuals of the control group in either speed or power of running and jumping.

### Correlation between physical activity and muscle strength

**Figure 3:**
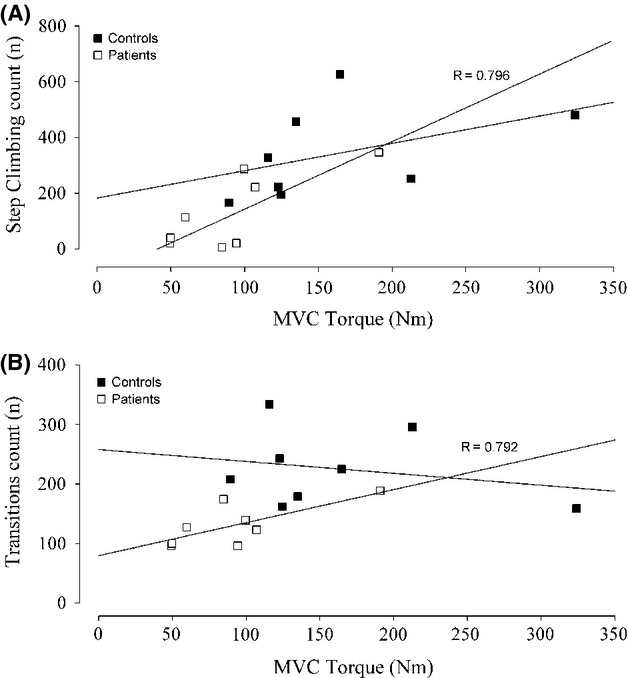
(A) Step-climbing count as a function of maximal isometric voluntary contraction (MVC) torque in patients and healthy individuals of the control group, and (B) transition count as a function of MVC torque in patients and healthy individuals of the control group. Linear regression curves are fitted through the data, and Pearson correlation coefficients are reported (*P* < 0.05).

Torque of knee extensor muscles recorded during isometric MVC was lower in CMT1A patients than healthy controls (91.93 ± 45.95 Nm and 161.03 ± 75.5 Nm, respectively). There was a significant correlation (*P* < 0.05) between MVC torque and number of steps climbed (Fig. 3A) and between MVC torque and number of transition (Fig. 3B) in CMT1A patients, whereas these correlations were not significant in the control group.

## Discussion

The main result of this study is that CMT1A patients showed a lower amount and intensity in some daily living activities with respect to the healthy individuals of the control group. Patients carried out a lower number of both ascending and descending steps and sit to stands, and selected a lower speed of walking and step climbing. Moreover, in CMT1A patients, the number of both ascending and descending steps and sit to stands was correlated with muscle strength.

From the analysis of generic physical activity indexes, such as total distance covered and number of steps performed during the whole day, it has been shown that CMT1A patients did not differ from healthy individuals of the control group. Moreover, both groups spent a similar amount of time in resting activities. Although there are no studies in the literature measuring daily living activities in CMT1A patients by means of inertial sensors, our results appear to be in contrast with previous observations of Aitkens and colleagues (Aitkens et al. 2005), based on self-reported levels of physical activity, which were lower in patients with various neuromuscular diseases (CMT, myotonic dystrophy, limb-girdle syndrome) compared to healthy individuals. This discrepancy could be ascribed not only to the inaccuracy of daily activity logs with respect to inertial sensors, but also to the heterogeneity of the patients' group, which may have included individuals with higher impairment in physical performances than our patients.

Even if CMT1A patients covered the same distance and performed the same number of steps as healthy controls, they carried out a lower number of both ascending and descending steps and performed a lower number of sit-to-stand and stand-to-sit transitions, which is one of the most innovative results of this study due to the inertial sensors' feature of discriminating specific changes in posture and body motions. It can be speculated that CMT1A patients avoid most demanding tasks requiring high-intensity contractions of the lower limbs muscles, both eccentric and concentric, as a consequence of their functional limits. Moreover, the low number of sit to stands means that CMT1A patients have a more sedentary lifestyle that could be one of the reasons to explain the decline in aerobic capacity reported in literature in patients with neuromuscular diseases (Wright et al. 1996; Fowler 2002; Kilmer 2002; El Mhandi et al. 2008).

With regard to physical exercise intensity, mean speed of walking and step climbing during the 24-h sessions was significantly lower in CMT1A patients with respect to healthy controls, which is in line with the results of other researchers who measured speed of walking in a laboratory environment (Kalkman et al. 2005; El Mhandi et al. 2008; Menotti et al. 2011). Nevertheless, this is the first observation measuring speed of walking and step climbing in CMT1A during spontaneous daily living activities, which is another major strength of this study. In healthy older individuals showing a decline in cardiovascular fitness, neuromuscular function, and functional abilities, all of which have been attributed to the combined effects of both aging and sedentary lifestyle, it has been demonstrated that it is not the amount but rather the intensity of daily living activities that correlates with these physiological factors (Laudani et al. 2013). Therefore, it can be argued that in CMT1A patients, who also show a decline in cardiovascular fitness, neuromuscular function, and functional abilities (Wright et al. 1996; Fowler 2002; Kilmer 2002; El Mhandi et al. 2008), this decline can be attributed not only to the effects of the disease itself but also to the low intensity at which daily living activities are carried out.

Estimates of daily energy expenditure showed that there were no differences between CMT1A patients and healthy individuals of the control group, with distance covered and time spent in walking activities being similar in the two groups. This result appears to be in contrast with previous observation by Menotti et al. (2011), who demonstrated that a homogeneous group of CMT1A patients have a greater energy cost of walking per unit of distance when compared with healthy individuals. Similarly, Aitkens et al. (2005) speculated that individuals with neuromuscular diseases have a low economy of movements by monitoring heart rate and self-reported daily living activities. Therefore, we expected to record a higher daily energy expenditure in CMT1A patients as they covered the same distance and spent the same time in walking activities with respect to the healthy controls. It is likely that this unexpected result can be attributed to the inaccuracy of the IDEEA device in estimating daily energy expenditure as it does not take into account the effects of altered walking patterns in CMT1A patients (Mazzaro et al. 2005; Don et al. 2007; Newman et al. 2007).

Charcot–Marie–Tooth 1A patients showed lower isometric strength of the knee extensor muscles with respect to healthy individuals, which is consistent with previous results of other authors (Lindeman et al. 1999; Kalkman et al. 2005). A novel finding of our study is the significant correlation between isometric strength and the number of both ascending and descending steps and sit to stands in the patients group. Therefore, not only do CMT1A patients carry out a lower number of both ascending and descending steps and sit to stands than the healthy individuals but also, among patients, they are the weakest individuals who actually perform the lowest number of these daily living activities. These correlations support the speculation that lower levels of muscle strength in patients could induce them to select and perform less demanding tasks during daily living activities.

In this study, the selection of a homogeneous sample represents a strength of this study, but it also brings forth a limitation as far as the generalization of the results. Even though the size of the sample is relatively small, both the number of both ascending and descending steps and sit to stands significantly correlated with muscle strength. This limitation can therefore be seen as a fruitful avenue for future research about the role of muscle strength on activities of daily living in CMT1A patients.

In conclusion, this research demonstrated that CMT1A patients differ from healthy individuals not only in the amount but also in the intensity of daily living activities. Moreover, in CMT1A patients, some demanding activities of daily living, such as stepping and sit to stand, correlated with muscle strength. As a practical application, it appears than CMT1A patients may benefit from strengthening lower limb muscles, particularly the knee extensors. Further studies should focus on the design of specific programs aimed at improving neuromuscular function in this group of patients.
